# Generational differences in clock drawing test performance

**DOI:** 10.1017/S1355617726101830

**Published:** 2026-02

**Authors:** Bluyé DeMessie, Ava Tsapatsaris, Leigh Rudberg, Simone Glajchen, Molly E. Zimmerman, Richard B. Lipton, Michael L. Lipton

**Affiliations:** 1 The Dominick P. Purpura Department of Neuroscience, Albert Einstein College of Medicine, Bronx, NY, USA; 2 New York University, New York, NY, USA; 3 Stern College for Women, Yeshiva University, New York, NY, USA; 4 Columbia University, New York, NY, USA; 5 Department of Psychology, Fordham University, Bronx, NY, USA; 6 The Saul R. Korey Department of Neurology, Albert Einstein College of Medicine; Department of Epidemiology & Population Health, Albert Einstein College of Medicine and Department of Psychiatry and Behavioral Sciences, Albert Einstein College of Medicine, Bronx, NY, USA; 7 Department of Radiology, Columbia University Vagelos College of Physicians and Surgeons, New York, NY, USA; 8 Department of Biomedical Engineering, Columbia University, New York, NY, USA

**Keywords:** Clock drawing test, age factors, analog literacy, neuropsychological assessment, cohort effect, cognitive testing

## Abstract

**Objective:**

The clock drawing test is widely used in clinical neurological and neuropsychological assessment. We hypothesized that younger adults would have greater problems with clock drawing than older adults, perhaps due to decreasing analog clock use.

**Methods:**

Cross-sectional study analyzing clock drawing performance and cognitive function across four generations (Gen Z, Millennials, Gen X, Baby Boomers). Participants included 92 adults divided into two generations (63 younger [18–42 years old] and 29 older [43–77 years old]) assessed between October 2022 and December 2024. Participants were screened to exclude conditions affecting cognition. The primary outcome was performance errors in clock drawing (e.g., writing “11:10” instead of drawing an analog clock, or placing hands incorrectly), assessed using standardized criteria. Cognitive function was assessed using eight computerized tests (CogState) measuring processing speed, attention, executive function, visuospatial memory, and verbal memory. Confirmatory factor analysis (CFA) validated three cognitive domain composites: Speed/Attention, Executive/Spatial, and Verbal Memory.

**Results:**

Performance errors were significantly more prevalent among younger participants compared with older participants (*p* = .016; risk ratio, 4.45). The effect size was large (Cohen’s *h* = .63). The generation effect was stronger (OR = 28.66, *p* = .003) after controlling for CFA-validated cognitive domain composites. This provides strong evidence that generational differences are independent of cognitive abilities.

**Conclusions:**

Younger adults demonstrate significantly higher rates of clock drawing errors compared with older adults, independent of cognitive performance. These findings suggest a need for generation-specific or age adjusted norms in clock drawing test interpretation.

## Statement of Research Significance

**Research Question(s) or Topic(s):** This study investigated whether younger adults have more difficulty with the clock drawing test compared to older adults, possibly due to decreased exposure to analog clocks in the digital age. **Main Findings:** Younger adults (ages 18–42) were over four times more likely to make errors on the clock drawing test compared to older adults (ages 43–77). Common errors included writing digital time (e.g., “11:10”) instead of drawing analog clock hands or incorrectly placing the hands. These difficulties were not associated with cognitive performance. **Study Contributions:** This study provides the first evidence that generational differences in clock drawing performance exist independently of cognitive function. The findings suggest that the clock drawing test, while still valuable for older adults, may need revised interpretation guidelines when used with younger, digital-native individuals in neurological assessments.

## Introduction

The Clock Drawing Test (CDT), introduced in 1915, is a widely used bedside neuropsychological screening tool, though its sensitivity for detecting mild impairment varies across studies and populations (Hazan et al., 2018). The test typically requires patients to draw a clock face with all numbers in correct sequence and set the hands to “ten after eleven”; a time selected for its cognitive complexity and sensitivity to frontal lobe impairment (Edith Kaplan, 1988; E. Kaplan, 1990). Successful completion requires intact functioning across distributed circuits: temporal lobes for semantic knowledge of clock properties, parietal regions for spatial organization and attention, and frontal systems for executive planning, response inhibition, and abstract thinking (Royall et al., 1999). Neuroimaging has demonstrated CDT performance correlates with activation patterns in the right parietal cortex and bilateral frontal regions, particularly during number placement and hand setting components (Cohen et al., 2014). These neural correlates suggest that the CDT may be sensitive to dysfunction in frontoparietal networks, potentially flagging areas of concern warranting further evaluation (Mainland & Shulman, 2017). Taking less than two minutes to complete and requiring no special forms or supplies, the CDT’s simplicity and capacity to provide preliminary insight into multiple cognitive domains makes it a valuable tool within broader clinical assessments (Aprahamian et al., 2009; Hazan et al., 2018).

Emerging evidence suggests CDT validity may be affected by exposure to technology and consequent diminished exposure to analog timekeeping devices, particularly among Digital-Native Adults (individuals born after 1980 who grew up with widespread access to digital technology) (Marc, 2001). Winstead et al. (2021) found that 28.6% of neurologically intact college students failed to achieve perfect CDT scores, while McDaniel et al. (2024) observed that 25% of young adults scored below expected ranges. This contrasts markedly with historical data showing near-perfect performance among educated young adults (Siciliano et al., 2016). Most recently, Vishnevsky et al. (2024) documented that Generation Z (born 1997–2003, study mean age 19.7 years) averaged only 8.1/10 points on the CDT despite cognitive performance that was otherwise within normal limits.

We hypothesized that younger adults, having grown up with time displays that are predominately digital, would demonstrate less proficiency with analog clock drawing compared to older adults who had greater exposure to analog clocks during their formative years. This experiential difference could manifest as increased errors in clock construction and time representation, independent of underlying cognitive abilities. Previous investigations of generational effects on CDT performance have been limited by small sample sizes and, crucially, lack of neuropsychological assessment of cognitive function (McDaniel et al., 2024; Vishnevsky et al., 2024; Winstead & Holman, 2021). No studies have directly compared performance across generational cohorts in concert with explicit measurement of cognitive performance. Our study addresses these limitations by examining CDT performance across four generations: Generation Z (born 1997–2012), Millennials (born 1981–1996), Generation X (born 1965–1980), and Baby Boomers (born 1946–1964), while incorporating standardized cognitive testing of processing speed, memory, executive function, and attention networks. We aimed to distinguish true generational differences from potential confounding by underlying variation in cognitive performance.

## Methods

### Study design and population

This cross-sectional study analyzed CDT performance across age cohorts, grouped into younger and older generations. Participants were recruited from prior studies of healthy individuals conducted by our research group examining cognitive function and neuroimaging correlates. The current study represents a focused secondary analysis specifically examining generational differences in CDT performance that were not addressed in the original studies. This study received approval from the Institutional Review Boards of both Albert Einstein College of Medicine and Columbia University and was conducted in accordance with the principles of the Helsinki Declaration. All participants provided written informed consent prior to participation.

Participants aged 18 years and older were selected by contacting participants in prior studies of healthy individuals. Those who agreed to enroll completed assessments between October 20, 2022, and December 5, 2024. The younger cohort included Generation Z (aged 18–26 years at testing) and Millennials (aged 27–42 years at testing), while the older cohort included Generation X (aged 43–58 years at testing) and Baby Boomers (aged 59–77 years at testing).

Inclusion criteria required participants to be 18 years or older with stated willingness to comply with study procedures and availability for the study duration. Exclusion criteria comprised history of neurological disorders (e.g., demyelinating disease, stroke), neurodevelopmental disorders, or major psychiatric disorders (e.g., schizophrenia, bipolar disorder); COVID-19 hospitalization requiring oxygen supplementation; active acute COVID-19 (defined as <14 days since diagnosis without symptom improvement or fever resolution within 48 hours); prior traumatic brain injury (based on the Ohio State University Traumatic Brain Injury Identification Screen); or new active medical problems that might interfere with cognitive assessment.

### Clock drawing test assessment

Following standardized CDT administration procedures (Pinto & Peters, 2009), each participant received identical verbal instructions: “Please draw a clock face, placing all the numbers on it and set the time to 10 past 11.” Three independent raters, blinded to participant age and study hypotheses, evaluated clock drawing performance using standardized Rouleau (1992) scoring criteria. The primary outcome measure was the presence of performance errors, which included two specific error types as defined by Rouleau, et al.: conceptual errors or stimulus-bound errors.

Conceptual errors were characterized by loss or deficit in accessing knowledge of the attributes, features, and meaning of a clock (loss of visuospatial knowledge about clock attributes, e.g., incorrect clock face), and were classified into two subtypes: (A) Misrepresentation of the clock itself (only a clockface without numbers or inappropriate use of numbers), suggesting the unavailability of a correct graphic representation of a clock; and (B) Misrepresentation of the time on the clock where hands are either absent or inadequately represented, or the time is written on the clock, suggesting a deficit in knowledge of the feature (the hands) that confers most of the meaning of a clock.

Stimulus-bound errors were specifically defined as the tendency of the drawing to be dominated or guided by a single stimulus (responses dominated by literal features of the instruction, e.g., drawing hands pointing to “10” and “11” when asked to set the time to 10 past 11), and were classified into two subtypes: (A) Setting the hands to point directly to 10 and 11 instead of recoding “10 past 11” to the correct minute hand position at 2, representing attraction to the strong stimulus source (“10”) rather than giving the appropriate response that involves a more complex operation; and (B) Writing the time in letters and/or numbers beside the “11” or between “10 and 11” on the clock, with hands either absent or pointed toward “10” and/or “11.” Per Rouleau et al., stimulus-bound Type B errors are classified as both stimulus-bound and conceptual because writing the time in words or numbers (rather than setting hands) indicates both literal interpretation of the instruction and fundamental misunderstanding of how analog clocks represent time. Consequently, individuals exhibiting this error type contribute to counts of both error categories.

The primary outcome was the presence of any performance error (binary: yes/no), defined as either conceptual deficits (Type A or B) OR stimulus-bound responses following Rouleau criteria. Participants were classified as demonstrating errors if they met criteria for either error type based on our grading system. We report the proportion of participants demonstrating any performance error. The two error types were combined into a single performance error measure based on theoretical considerations established before data analysis to capture fundamental difficulties with analog clock representation.

### Cognitive assessment

Cognitive performance was evaluated using the CogState computerized test battery, a standardized and validated assessment tool (CogState, 2020; Maruff et al., 2009). The battery comprised eight tests using developer-validated primary outcome measures (CogState, 2020; Falleti et al., 2003; Falleti et al., 2006; Maruff et al., 2009). Processing Speed was assessed using the Groton Maze Chase Test (GMCT), in which participants track a moving target around a 10 × 10 grid. The primary outcome measure was moves per second, with higher scores indicating better performance. Attention was assessed using two tasks. The Identification Task (IDN) is a choice reaction time task requiring participants to indicate whether a displayed playing card is red or black. The One-Back Task (ONB) requires participants to indicate whether the current card matches the immediately preceding card. For both tasks, the primary outcome measure was log_10_ transformed reaction time for correct responses, with lower scores indicating better (faster) performance. Working Memory was assessed using two tasks. The Groton Maze Learning Test (GMLT) is a hidden pathway maze task in which participants learn a 28-step pathway through a 10 × 10 grid across five consecutive trials. The primary outcome measure was total errors, with lower scores indicating better performance. The Two-Back Task (TWOB) requires participants to indicate whether the current card matches the card presented two trials earlier. The primary outcome measure was arcsine transformed proportion correct, with higher scores indicating better performance. Memory was assessed using three tasks. The International Shopping List Test (ISL) is a verbal list learning task in which participants recall 12 items across three learning trials. The International Shopping List Test-Delayed Recall (ISRL) assesses recall of the same list following a delay. For both verbal memory tasks, the primary outcome measure was total correct responses, with higher scores indicating better performance. The Groton Maze Learning Test-Delayed Recall (GMR) assesses spatial memory through recall of the previously learned maze pathway following a delay. The primary outcome measure was total errors, with lower scores indicating better performance.

### Statistical analysis

All analyses were performed using R version 4.2. The primary outcome was presence of CDT performance error, operationalized as a binary variable. Between-group comparisons employed Fisher’s exact test, selected *a priori* given anticipated sparse contingency table cells. Effect size was quantified using Cohen’s *h*. Concordance among the three independent raters was quantified using Fleiss’ κ.

To test whether generational differences were independent of cognitive performance, we employed a composite cognitive domain approach to address events-per-variable (EPV) limitations. The validity of the hypothesized domain structure was evaluated using confirmatory factor analysis (CFA). Prior to model estimation, all cognitive test scores were standardized to *z*-scores using sample means and standard deviations. Reaction time measures (IDN, ONB) and error counts (GMLT, GMR) were reversed (multiplied by −1) such that higher values uniformly indicate superior performance across all indicators, consistent with developer guidelines (CogState, 2020).

CFA models were estimated via maximum likelihood with robust standard errors (MLR estimator) and Satorra-Bentler scaled test statistics, which provide asymptotically correct parameter estimates and chi-square values under conditions of multivariate non-normality. The general *k*-factor measurement model is expressed as: *x* = Λη + ϵ, where *x* represents the *p* × 1 vector of observed cognitive test scores, Λ represents the *p* × *k* matrix of factor loadings, *η* represents the *k* × 1 vector of latent cognitive factors, and ϵ represents the *p* × 1 vector of measurement errors assumed uncorrelated across indicators.

Model fit was evaluated according to established criteria (Hu & Bentler, 1999): Comparative Fit Index (CFI), Tucker-Lewis Index (TLI), Root Mean Square Error of Approximation (RMSEA), and Standardized Root Mean Square Residual (SRMR). Acceptable model fit was defined *a priori* as CFI ≥ .90, TLI ≥ .90, RMSEA ≤ .10, and SRMR ≤ .08. For comparison of nested models, Bayesian Information Criterion (BIC) was employed; differences exceeding 10 units constitute strong evidence favoring the model with lower BIC (Raftery, 1995). Discriminant validity between latent factors was evaluated via inspection of inter-factor correlation estimates; correlations exceeding .85 indicate inadequate discrimination between constructs (Kline, 2015). Internal consistency reliability of the empirically validated composites was assessed using Cronbach’s coefficient α.

Following CFA validation, composite scores for each empirically supported cognitive domain were computed as the unweighted arithmetic mean of constituent standardized test scores. To evaluate whether cognitive performance accounted for generational differences in CDT deficit, two nested logistic regression models were specified:

Model 1 (demographic covariates): logit[*P*(*Y* = 1)] = β_0_ + β_1_(Generation) + β_2_(Sex) + β_3_(Education)

Model 2 (demographic and cognitive covariates): logit[*P*(*Y* = 1)] = β_0_ + β_1_(Generation) + β_2_(Sex) + β_3_(Education) + Σ_j_γ_j_(Cognitive Domain_j_)

where *Y* = 1 denotes presence of CDT performance error, Generation is coded 0 = Older and 1 = Younger, and the summation extends over the CFA-validated cognitive domain composites. The coefficient β_1_ quantifies the generation effect on the log-odds scale. Comparison of β_1_ between models tests whether cognitive abilities confound the generation-CDT association: attenuation of β_1_ in Model 2 relative to Model 1 would indicate confounding, whereas maintenance or strengthening would indicate independence of the generation effect from measured cognitive performance.

Standard assumptions for logistic regression were evaluated: binary outcome structure, independence of observations, absence of multicollinearity among predictors (variance inflation factors), and absence of unduly influential observations (Cook’s distance). Parameter estimates were obtained via maximum likelihood; odds ratios and corresponding 95% confidence intervals were derived by exponentiation of coefficients and Wald-based confidence limits.

## Results

### Study population

The analysis included 92 participants (63 younger, 29 older). Descriptive statistics for all variables are presented in Tables 1–4. Sample characteristics, cognitive test performance, and CDT error rates are detailed in the tables with means, standard deviations, proportions, and p-values. Demographic and clinical characteristics are shown in Table 1. Standardized clinical neurological examination revealed no abnormality in any participant. Brain MRI showed no visible structural abnormality in any participant. Inter-rater reliability for detection of CDT errors was excellent (Fleiss’ *κ* = .84).

**Table 1.** Demographic and clinical characteristics of study participants by age cohort

Characteristic	Gen Z (*n* = 5)	Millennial (*n* = 58)	Gen X (*n* = 26)	Boomer (*n* = 3)	Younger (*n* = 63)	Older (*n* = 29)	*P* Value
Age, mean (SD), y	26.9 (0.6)	33.4 (4.1)	51.2 (4.6)	67.9 (5.2)	32.9 (4.3)	52.9 (6.9)	< .001
Sex, no. (%)							> 0.99
F	2 (40.0%)	26 (44.8%)	11 (42.3%)	2 (66.7%)	28 (44.4%)	13 (44.8%)	
M	3 (60.0%)	32 (55.2%)	15 (57.7%)	1 (33.3%)	35 (55.6%)	16 (55.2%)	
Education, mean (SD), y	17.0 (4.9)	16.7 (4.0)	15.5 (5.0)	15.3 (1.2)	16.7 (4.0)	15.4 (4.8)	0.21
Race, no. (%)							0.89
American Indian/Alaska native	0 (0.0%)	1 (1.7%)	0 (0.0%)	0 (0.0%)	1 (1.6%)	0 (0.0%)	
Asian	0 (0.0%)	6 (10.3%)	2 (7.7%)	0 (0.0%)	6 (9.5%)	2 (6.9%)	
Black/African American	2 (40.0%)	17 (29.3%)	12 (46.2%)	1 (33.3%)	19 (30.2%)	13 (44.8%)	
More than one race	1 (20.0%)	3 (5.2%)	2 (7.7%)	0 (0.0%)	4 (6.3%)	2 (6.9%)	
Not reported	0 (0.0%)	2 (3.4%)	0 (0.0%)	0 (0.0%)	2 (3.2%)	0 (0.0%)	
Unknown	1 (20.0%)	2 (3.4%)	1 (3.8%)	0 (0.0%)	3 (4.8%)	1 (3.4%)	
White	1 (20.0%)	27 (46.6%)	9 (34.6%)	2 (66.7%)	28 (44.4%)	11 (37.9%)	
Ethnicity, no. (%)							0.31
Hispanic or latino	2 (40.0%)	16 (27.6%)	5 (19.2%)	0 (0.0%)	18 (28.6%)	5 (17.2%)	
Not hispanic or latino	3 (60.0%)	42 (72.4%)	20 (76.9%)	3 (100.0%)	45 (71.4%)	23 (79.3%)	
Charlson comorbidity index, mean (SD)	0.0 (0.0)	0.1 (0.3)	0.0 (0.2)	0.3 (0.6)	0.1 (0.3)	0.0 (0.2)	0.41
Comorbidity count, mean (SD)	0.4 (0.5)	0.4 (0.7)	0.3 (0.6)	0.7 (1.2)	0.4 (0.7)	0.3 (0.6)	0.73
Depression, no. (%)	0 (0.0%)	2 (3.4%)	2 (7.7%)	0 (0.0%)	2 (3.2%)	2 (6.9%)	0.59
Anxiety, no. (%)	1 (20.0%)	8 (13.8%)	2 (7.7%)	0 (0.0%)	9 (14.3%)	2 (6.9%)	0.49
Cardiovascular,no. (%)	0 (0.0%)	2 (3.4%)	1 (3.8%)	0 (0.0%)	2 (3.2%)	1 (3.4%)	> 0.99
Hypertension, no. (%)	0 (0.0%)	1 (1.7%)	2 (7.7%)	0 (0.0%)	1 (1.6%)	2 (6.9%)	0.24
Diabetes, no. (%)	0 (0.0%)	2 (3.4%)	0 (0.0%)	0 (0.0%)	2 (3.2%)	0 (0.0%)	> 0.99
Asthma, no. (%)	1 (20.0%)	7 (12.1%)	3 (11.5%)	0 (0.0%)	8 (12.7%)	3 (10.3%)	> 0.99

*Note:* Younger = Gen Z and Millennials (born 1981+); Older = Gen X and Boomers (born 1946–1980). Gen Z = born 1997–2012; Millennials = born 1981–1996; Gen X = born 1965–1980; Boomers = born 1946–1964.

**Table 2.** CDT performance statistics

Generation	Sample size	Number with deficit	Proportion (%)	95% CI
Younger	63	19	30.2	19.2–43.0
Older	29	2	6.9	0.8–22.8

**Table 3.** Error type breakdown

Errortype	Younger N (%)	Older N (%)
Conceptual deficit	18 (28.6)	2 (6.9)
Stimulus-bound error	11 (17.5)	1 (3.4)
Either error type	19 (30.2)	2 (6.9)

**Table 4.** Cognitive test performance (Raw scores) by generation

Cognitive test	Younger mean (SD)	Older mean (SD)	*P* value
GMCT (processing speed)	1.65 (0.27)	1.29 (0.32)	< 0.001
GMLT (executive)	51.5 (15.8)	65.0 (23.5)	0.002
GMR (memory)	6.8 (3.5)	10.0 (5.8)	0.003
IDN (attention)	2.68 (0.06)	2.73 (0.10)	0.007
ISL (memory)	28.8 (3.2)	26.1 (4.4)	0.002
ISRL (memory)	10.6 (1.6)	9.0 (2.4)	< 0.001
ONB (attention)	2.82 (0.13)	2.92 (0.14)	0.001
TWOB (memory)	1.22 (0.12)	1.12 (0.16)	0.002

*Note:* All scores are raw values without age-based standardization.

### Primary outcome

Performance errors (defined as presence of either conceptual deficits or stimulus-bound errors) were significantly more prevalent in the younger cohort: 19/63 (30.2%, 95% CI [19.2–43.0%]) compared to the older generation: 2/29 (6.9%, 95% CI [0.8–22.8%]), Fisher’s exact test *p* = .016, Chi-square test *p* = .028. The risk ratio was 4.37, with a medium-large effect size (Cohen’s *h* = .63). Detailed CDT performance statistics are shown in Table 2, and error type breakdown in Table 3.

**Figure 1.**
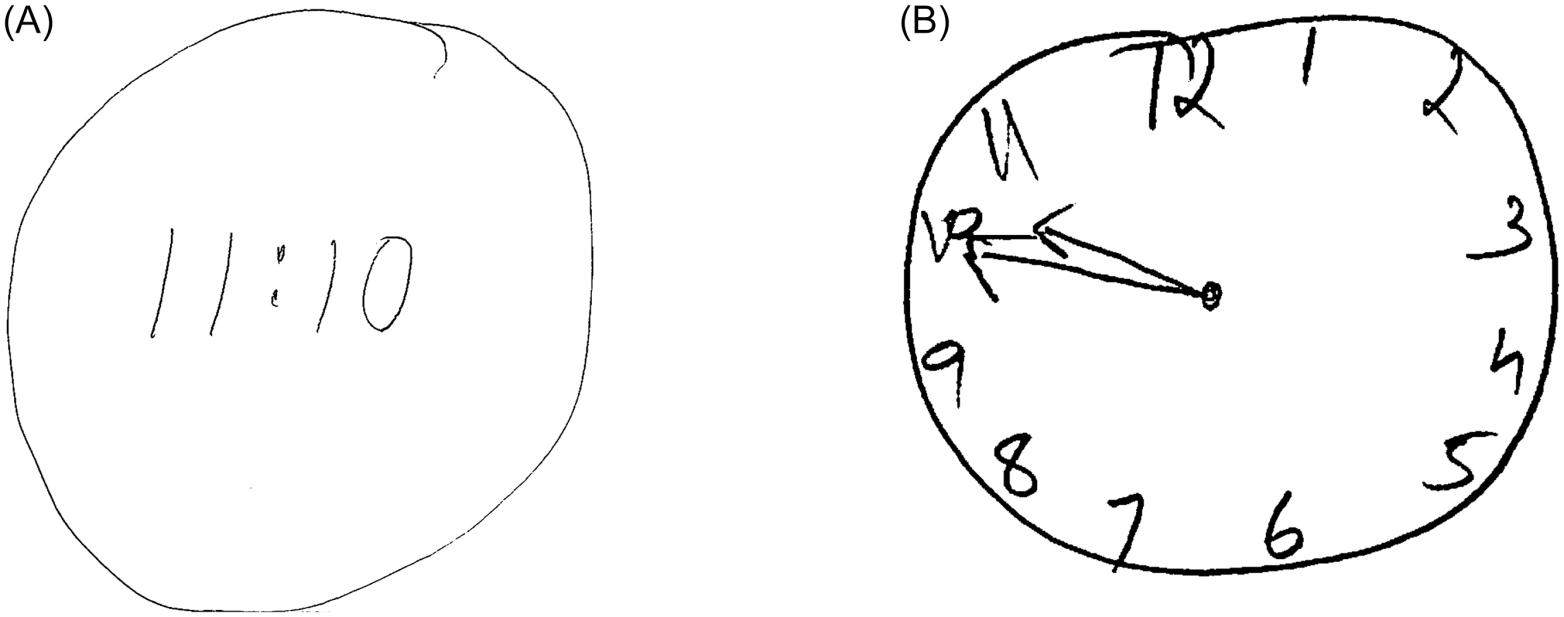
Examples of clock drawing test performance errors in digital-native adults. Panel A shows a participant’s digital-format response when instructed to “Please draw a clock face, placing all the numbers on it and set the time to 10 past 11.” Instead of drawing an analog clock face with hands, the participant wrote “11:10”, demonstrating a conceptual error in analog clock representation. Panel B illustrates a stimulus-bound response to the same instructions, where a different participant drew a clock face but placed the hands pointing directly to 10 and 11, failing to recode “10 past 11” to the correct hand positions (minute hand at 2, hour hand just past 11). This type of stimulus-bound error reflects the literal numbers in the instruction (“10” and “11”), and that the participant did not convert their meaning to their appropriate spatial positions on the analog clock face.

When broken down by specific generational cohorts, the data revealed a clear gradient: Gen Z showed the highest proportion of errors (40.0%, *n* = 5), followed by Millennials (28.8%, *n* = 58), Gen X (11.0%, *n* = 26), and Boomers (0%, *n* = 3). Representative examples of performance errors observed in Digital-Native Adults included drawing digital time displays and stimulus-bound responses where numbers were placed sequentially rather than in correct spatial positions (Figure 1). The risk ratio was 4.45 (95% CI [1.10, 17.95]), indicating that younger participants were more than four times as likely to demonstrate performance errors compared with older participants. The effect size (Cohen’s *h*) was −.71 (95% CI [−1.19, −.22]), representing a large generational difference.

Specific error types are detailed in Table 3. Within the younger cohort, specific error types identified included conceptual deficit type A errors (5[7.8%]), characterized by misrepresentation of the clock itself, and conceptual deficit type B errors (13[20.3%]), characterized by misrepresentation of the time on the clock. Stimulus-bound errors were also observed, with type A errors (5[7.8%]) where hands were set for 10 to 11 instead of 10 past 11, and type B errors (10[15.6%]) where the time was written in numbers/letters between 10 and 11. In contrast, the older cohort exhibited only conceptual deficit type B errors (2[6.9%]) with no occurrences of other error types. The overall performance error prevalence was markedly higher in the younger cohort (19[29.7%]) compared with the older cohort (2[6.9%]).

Qualitative analysis revealed younger participants with poor performance demonstrated high confidence despite significant errors, showing no awareness of deficiencies while maintaining conviction in the accuracy of fundamentally flawed clock depictions.

### Cognitive analysis

To determine whether these generational differences reflected underlying cognitive dysfunction or experiential factors related to analog clock exposure, we examined associations between composite cognitive domain scores and CDT error. Descriptive statistics for all cognitive measures are presented in Table 4. Consistent with the age differential between cohorts, multiple cognitive tests demonstrated significant between-group differences when analyzed using raw, unstandardized scores (all *p* < .01).

CFA was conducted and a three-factor model demonstrated acceptable fit: χ^2^ *SB*(17) = 34.82, CFI = .94, TLI = .90, RMSEA = .11 (90% CI [.05, .16]), SRMR = .08. The validated factor structure comprised: (1) Speed/Attention, with indicators GMCT (*λ* = .65), IDN (*λ* = .88), and ONB (*λ* = .89); (2) Executive/Spatial, with indicators GMLT (*λ* = .92), GMR (*λ* = .89), and TWOB (*λ* = .59); and (3) Verbal Memory, with indicators ISL (*λ* = .73) and ISRL (*λ* = .93). All standardized factor loadings exceeded .50 and were statistically significant (*ps* < .001). Inter-factor correlations ranged from *r* = .37 (Executive/Spatial–Verbal Memory) to *r* = .49 (Speed/Attention–Executive/Spatial), all *ps* < .002, supporting discriminant validity (all *rs* < .85). Internal consistency was adequate for all composites: Speed/Attention (Cronbach’s *α* = .84), Executive/Spatial (*α* = .83), and Verbal Memory (*α* = .81).

### Generation-cognition independence

The unadjusted generation effect (Model 1) was statistically significant (OR = 5.96, 95% CI [1.26, 28.28], Wald χ^2^(1) = 5.02, *p* = .025), indicating that younger adults demonstrated approximately six-fold higher odds of CDT deficit relative to older adults. Following adjustment for the three cognitive domain composites (Model 2), the generation effect strengthened substantially (OR = 28.66, 95% CI [3.05, 269.42], Wald χ^2^(1) = 8.86, *p* = .003). Strengthening rather than attenuation upon cognitive adjustment constitutes a suppression effect: younger adults exhibit superior cognitive performance on average, yet demonstrate elevated CDT deficit rates. Once this cognitive advantage is statistically controlled, the generational disparity becomes more pronounced.

Among the cognitive domain composites in Model 2, Speed/Attention demonstrated a significant protective association with CDT deficit (OR = .37, 95% CI [.17, .81], Wald χ^2^(1) = 6.28, *p* = .012), whereas Executive/Spatial (OR = .53, 95% CI [.23, 1.24], Wald χ^2^(1) = 2.15, *p* = .14) and Verbal Memory (OR = 1.98, 95% CI [.89, 4.43], Wald χ^2^(1) = 2.79, *p* = .095) did not attain statistical significance. Model diagnostics confirmed absence of multicollinearity (all VIF <2.5) and no unduly influential observations (all Cook’s *D* <.5).

## Discussion

In this study of generational differences in CDT performance, we found younger adults were significantly more likely to demonstrate performance errors compared with older adults, with over a 4-fold increased risk. These performance errors manifested in two primary forms: digital-format substitutions (e.g., writing “11:10” instead of drawing an analog clock face with hands) and stimulus-bound responses (e.g., drawing a clock face but incorrectly placing hands pointing directly to 10 and 11, rather than positioning the minute hand at 2 and the hour hand just past 11 to represent “10 past 11”). These errors reflect a fundamental misunderstanding of analog clock representation or an inability to translate verbal time instructions into their correct spatial positions on an analog clock face.

As a cognitive screening tool, the CDT demonstrates limited sensitivity to individual differences in cognitive domains within healthy populations. This is an important consideration for interpreting our findings. The modest cognitive associations observed in our sample likely reflect the restricted range of cognitive performance in healthy adults rather than a true absence of cognitive contributions to CDT performance in clinical populations. Our study was not powered to detect subtle cognitive influences within the normal range. However, the critical finding is that within-generation cognitive variations (including education level, which did not differ between generations) do not fully account for the robust between-generation differences in CDT performance. Even when controlling for all measured cognitive abilities, the generational effect remains highly significant, suggesting that experiential factors rather than cognitive or educational differences drive the observed generational gap.

Our findings align with broader societal trends in timepiece use and technological adaptation (Marc, 2001; McDaniel et al., 2024; Vishnevsky et al., 2024; Winstead & Holman, 2021). The higher prevalence of performance errors in younger generations may therefore reflect reduced exposure to analog time formats, rather than cognitive dysfunction. The younger cohort came of age during rapid technological transition. Smartphones became ubiquitous in the late 2000s, providing constant digital time displays (Pew Research Center, 2024). Many younger adults report rarely using analog clocks – checking time on phones, computers, digital displays rather than wall clocks or watches (YouGov Survey, 2025). We suggest that the clock drawing test, while still valuable for older adults, may need revalidation for younger populations as a neurological assessment tool.

Our findings reveal a significant generational discrepancy in CDT performance that appears independent of cognitive function, with younger participants showing markedly higher rates of performance errors despite equivalent or superior cognitive performance. These findings are most parsimoniously explained by differential familiarity with analog clock representations across generations. Observations of other studies on the CDT in younger individuals include Vishnevsky et al. (2024) who reported Generation Z adults averaged 8.1/10 points on the CDT and Winstead et al. (2021) who found nearly 30% of college students failed to achieve perfect scores. We extend these findings by demonstrating that CDT performance differences persist even when controlling for function across multiple cognitive domains. Our findings contrast starkly with historical reports: studies from the 1990s consistently showed near-perfect CDT performance among educated young adults (Freedman, 1994).

Our findings have important implications for clinical neurological practice, which routinely employs the CDT. The substantial generational difference in CDT performance suggests age-based norms may be inadequate, and generation-specific norms may be necessary for accurate assessment of the CDT.

### Limitations

Several limitations warrant consideration. Our sample size, while adequate for detecting the large effect sizes observed, may limit power to detect smaller associations or interactions between variables. However, the robustness of our findings, despite unequal sample sizes across generations and modest statistical power, suggest the findings may underestimate the true effect. Our findings derive from one geographic region (the greater New York City area) and may not generalize to populations which differ, for example, by the timing on which digital timekeeping technology was adopted and the rate and degree to which analog timekeeping was displaced. Additionally, the cross-sectional design cannot fully disentangle generational effects from potential cohort effects or developmental trajectories. Future studies with larger, more geographically diverse samples and longitudinal designs would strengthen these findings. The study lacked a direct measure of analog clock exposure which might have facilitated a direct test of the hypothesis. Further investigation is needed to validate our findings in larger, more balanced samples and to explore whether similar generational effects exist for other visuospatial tasks. Additionally, development of alternative scoring systems that account for generational differences in visuospatial processing patterns could enhance the test’s clinical utility across age groups.

## Conclusions

CDT performance errors as a function of younger generation are not explained by cognitive performance across multiple domains. Validation of the CDT for younger digital-native individuals is needed.
